# Remifentanil Preconditioning Attenuates Hepatic Ischemia-Reperfusion Injury in Rats via Neuronal Activation in Dorsal Vagal Complex

**DOI:** 10.1155/2018/3260256

**Published:** 2018-05-10

**Authors:** Cui Cui, Fang Yu, Suqing Yin, Yuting Yang, Yingfu Jiao, Chiwai Cheung, Xiaomin Wang, Bo Qi, Yaling Liu, Peiying Li, Weifeng Yu, Jie Xiao, Liqun Yang

**Affiliations:** ^1^Department of Anesthesiology, Eastern Hepatobiliary Surgery Hospital, the Second Military Medical University, Shanghai, China; ^2^Department of Anesthesiology, Renji Hospital, School of Medicine, Shanghai Jiao Tong University, Shanghai, China; ^3^Department of Anesthesiology, The University of Hong Kong, Pokfulam, Hong Kong; ^4^Laboratory and Clinical Research Institute for Pain, Department of Anaesthesiology, The University of Hong Kong, 21 Sassoon Road, Pokfulam, Hong Kong

## Abstract

Remifentanil, an ultra-short acting opiate, has been reported to protect against hepatic ischemia-reperfusion injury, which is a major cause of postoperative liver dysfunction. The objective of this study was to determine whether a central vagal pathway is involved in this protective procedure. Rat models of hepatic ischemia-reperfusion were used in the experimental procedures. The results revealed that intravenous pretreatment with remifentanil decreased serum aminotransferases and hepatic histologic damage; however, an intraperitoneal injection of *μ*-opioid receptor antagonist did not abolish the protection of remifentanil preconditioning. c-Fos immunofluorescence of the brain stem showed that dorsal motor nucleus of the vagus was activated after remifentanil preconditioning. Moreover, serum alanine aminotransferase, histopathologic damage, and apoptosis decreased in remifentanil preconditioning group compared to vagotomized animals with remifentanil preconditioning, and there was no statistical difference of TNF-*α* and IL-6 between NS/Va and RPC/Va groups. In addition, remifentanil microinjection into dorsal vagal complex decreased serum aminotransferases, inflammatory cytokines, and hepatic histologic injury and apoptosis, and these effects were also abolished by a peripheral hepatic vagotomy. In conclusion, remifentanil preconditioning conferred liver protection against ischemia-reperfusion injury, which was mediated by the central vagal pathway.

## 1. Introduction

Hepatic ischemia-reperfusion injury (IRI) is one of the most common complications associated with liver transplantation, hepatic lobectomy, massive trauma, or hemorrhagic shock [1–3], which is unavoidable and usually leads to postoperative liver failure [4, 5]. Studies showed that there are multiple molecules participating in hepatic IRI, such as reactive oxidant species (ROS), HMGB1, and TLR4 [4], as well as the mitochondrial dysfunction and autophagy [6]. Both ischemic preconditioning (IPC) and remote ischemic preconditioning (RIPC) had been identified to improve the outcome of organs suffering from IRI [7, 8]. Opioid receptors even an extrahepatic opioid receptor-associated mechanism may participate in this protection [9, 10].

Remifentanil, an ultra-short acting and nonspecific esterase-metabolized opioid receptor agonist, is one of the most widely used opioids because of its titratable pharmacokinetic profile that allows high intraoperative dosing without any delay in recovery [11]. Furthermore, there was an increasing body of literature showing that remifentanil protects against hepatic IRI in animal models, which is probably mediated by activating p38MAPK signal pathway [12], modulating the hepatic IL-18/IL-18 binding protein balance, inhibiting IL-18 signal pathway [13], and activating antiapoptotic pathways [14]. Our previous research has indicated that remifentanil preconditioning (RPC) reduces hepatic ischemia-reperfusion injury in rats via inducible nitric oxide synthase expression [15]. In addition, by virtue of its molecular structure, remifentanil circulates primarily in the nonionised moiety which helps it to penetrate the liposoluble blood-brain barrier (BBB) and to equilibrate across the plasma/effect site interface [16]. It combines to the opioid receptors in the central nervous system (CNS) and exerts its pharmacological roles immediately after intravenous administration [17]. Therefore, a potential central pathway by which remifentanil exerts liver protection following IRI was considered.

Dorsal vagal complex (DVC) was considered as the central of the vagus nerve, the tenth cranial nerve, and interfaces with parasympathetic control of major organs of the human body [18], including dorsal motor nucleus of the vagus (DMV), nucleus tractus solitarius (NTS), and area postrema (AP). The vagus nerve sets links between CNS and peripheral organs, regulates metabolic homeostasis, and constitutes a major component of inflammatory reflex, which modulates innate immune responses during infection and tissue injury [19–21]. It has also been revealed to attenuate hepatocyte apoptosis after ischemia-reperfusion [22] by activating *α*7 nicotinic acetylcholine receptor [23], which may be due to inhibition of the inflammatory response through HO-1 induction [24].

Therefore, our study was designed to investigate whether RPC protects against hepatic IRI via activation of *μ*-opioid receptor in CNS and if so, whether the vagus nerve participates in this procedure.

## 2. Materials and Methods

### 2.1. Animals

All experiments were carried out on adult male Sprague-Dawley rats (240–280 g) obtained from the Animal Care and Use Committee of the Second Military Medical University (Shanghai, China). Animals were kept at room temperature (22–25°C), subjected to a standard light-dark cycle, and allowed for free access to food and water until the night before the surgical procedure.

### 2.2. Hepatic Ischemia-Reperfusion Surgical Preparation

Anesthesia was induced by intraperitoneal injection of pentobarbitone (60 mg·kg^−1^, Dainabot, Osaka, Japan) and maintained by repeated doses of 25 mg·kg^−1^ if necessary, primarily based on animal movements. All the surgical procedures were performed under sterile conditions. After tracheotomy and tracheal intubation, mechanical ventilation was maintained with atmosphere air at 60–70 breaths·min^−1^, using a Harvard Apparatus Rodent Respirator (Harvard Apparatus, Boston, MA). Body temperature was controlled by using a heating pad placed under the animal to keep the rectal temperature at 36 ± 1°C. Laparotomy was carried out through a midline incision, the ligamentous attachments from the liver to the diaphragm were severed, and the liver was exposed. Ischemia of the median and left lateral lobes of the liver was produced by clamping the corresponding vascular pedicle containing the portal vein and branches of the hepatic artery with an atraumatic microvascular clamp for 45 min. Other hepatic lobes were not managed during the procedure. At the end of the ischemia period, the vascular clamp was removed, and the liver was perfused for 2 h. After reperfusion, 3–5 ml of blood in the vena cava was collected by sterile syringes without anticoagulant and centrifuged to separate the serum. The serum samples were stored at −20°C for aminotransferase analysis. Then the liver was perfused with cold saline through the portal vein. Ischemic left hepatic tissue samples were collected, and the specimens were fixed in 4% paraformaldehyde and embedded in paraffin for histologic studies. The animals were given an additional dose of pentobarbitone (50 mg) before being sacrificed by exsanguination.

### 2.3. Study Groups and Experimental Protocol

**Figure 1:**
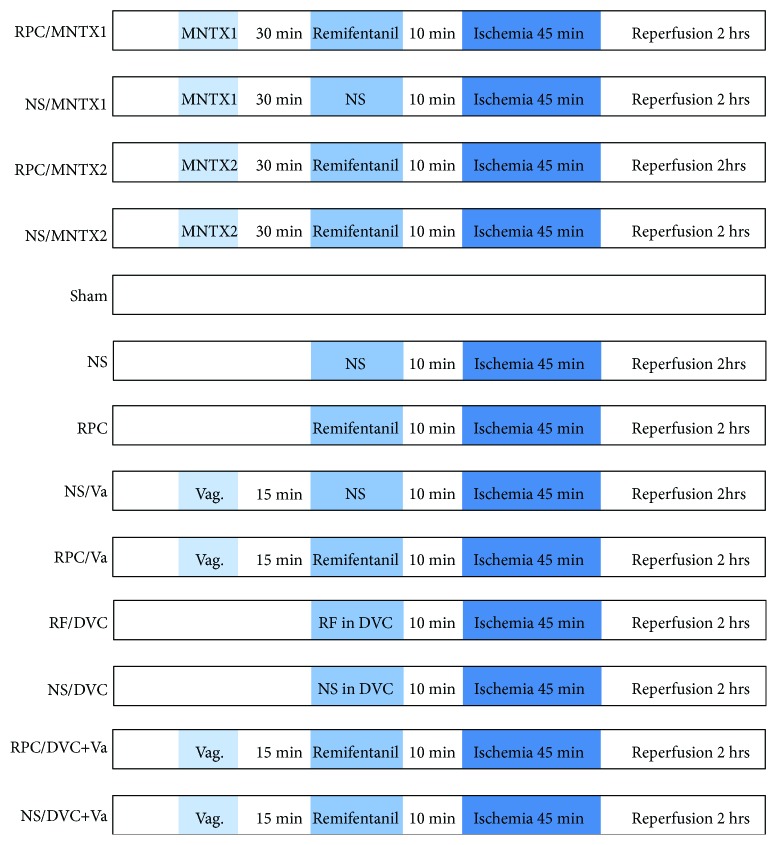
The protocols followed for all series of experiments are presented as listed. RPC: remifentanil preconditioning; NS: normal saline; MNTX1: 0.5 mg·kg^−1^ of MNTX; MNTX2: 5 mg·kg^−1^ of MNTX; Va: vagotomy; RF: remifentanil; DVC: dorsal vagal complex.

The study was carried out with three series of experiment as below (Figure 1):

First of all, the experiment was designed to determine whether RPC protects the liver against IRI by the activation of central *μ*-opioid receptors, and animals were randomly assigned to receive one of the following five treatments (*n* = 8 in each treatment group and *n* = 5 in the sham group, Figure 1(a)). Methylnaltrexone (MNTX) is one of the latest agents of peripherally acting *μ*-opioid antagonists which cannot cross the hematoencephalic barrier and was considered to counteract the side effects of opioids:

1. NS/MNTX1 group: rats were pretreated with intraperitoneal injection of MNTX in 0.5 mg·kg^−1^, followed by an intravenous injection of normal saline (1 Ml·min^−1^) within 15 minutes. The HIR was the same as described above.
2. RPC/MNTX1 group: rats were intraperitoneally injected with 0.5 mg·kg^−1^ of MNTX, followed by an intravenous injection of 1 Ml·min^−1^ remifentanil titrated by a syringe pump (2 *μ*g·kg^−1^·min^−1^) within 15 minutes. HIR was induced 10 minutes after remifentanil administration with ischemia for 45 minutes and 120 minutes reperfusion.
3. NS/MNTX2 group: rats were pretreated with intraperitoneal injection of 5 mg·kg^−1^ of MNTX, and the other procedures were the same as those in NS/MNTX1 group.
4. RPC/MNTX2 group: the dosage of MNTX injected into the peritoneal cavity was increased to 5 mg·kg^−1^. Other procedures were the same as those in RPC/MNTX1 group.
5. Sham group: Animals were performed with laparotomy and dissection of the portal vein but not clamping.

Subsequently, to investigate whether the vagus nerve is involved in RPC against hepatic IRI, a selective hepatic vagotomy was performed and the surgical procedure was the same as described previously [25]. A retrograde labelling of the vagus nerve was achieved with 1,1′-dioctadecyl-3,3,3′,3′-tetramethylindocarbocyanine perchlorate (Dil, Life Technologies Corporation, CA, USA), a fluorescent tracer, which was injected into the liver by a microinjector, to rule out an incomplete vagotomy. The inferior ganglion of the vagus nerve was dissected and analyzed with an immunofluorescence microscopy after 2 weeks of Dil injection. The rats were randomly assigned into the following 4 groups (*n* = 8 in each group, Figure 1(b)):

1. NS/Va group: the vagotomized animals were preconditioned with intravenous injection of normal saline (1 Ml·min^−1^) within 15 minutes and experienced IR surgical procedure.
2. RPC/Va group: the vagotomized animals underwent the RPC and HIR as described in our previous study [11].
3. NS control: rats were preconditioned with the same dose of normal saline as NS/Va group before HIR.
4. RPC group: rats were preconditioned with the same dose of remifentanil as RPC/Va group before HIR.

Furthermore, to explore whether the DVC is the exact central regulator by which RPC exerts its protection against hepatic IRI, and whether the hepatic vagus nerve is the crucial pathway participate in that, the third part of our study was primarily carried out with the following groups (*n* = 8 in each group, Figure 1(c)):

1. NS/DVC group: 1 *μ*L of normal saline was microinjected into DVC, followed by hepatic IR procedure.
2. RF/DVC group: the microinjection of 1-microgram remifentanil for 1 *μ*L into the DVC was performed, followed by HIR procedure.
3. NS/DVC + Va group: the microinjection of 1 *μ*L normal saline into the DVC was carried out on the vagotomized rats, followed by the HIR procedure.
4. RF/DVC + Va group: the microinjection of 1-microgram remifentanil for 1 *μ*L into the DVC was carried out on the vagotomized rats, followed by the HIR procedure.

### 2.4. Measurement of Serum Aminotransferase

Serum alanine aminotransferase (ALT) and aspartate aminotransferase (AST) were measured using commercial available kits (Nanjing Jiancheng Biochemicals Ltd., China) with a Model 7600 autobiochemistry analyzer (Hitachi, Tokyo, Japan).

### 2.5. Histopathology Examination and Apoptotic Cell Detection

All the histopathology examinations were performed by a pathologist who was blinded to the grouping to identify the underlying necrotic and apoptotic statuses of the liver. Liver samples were excised from the anterior edge of the left lobe 2 hours after reperfusion. Small slices of about 0.5 cm × 0.5 cm were fixed immediately in 4% buffered paraformaldehyde (pH = 7.2). These portions were cut into 4-micrometer-thick sections and stained with hematoxylin and eosin (H&E). High-powered microscopy (magnification: 200x) was used to examine these sections for signs of liver injury, such as condensation of nuclei (nuclear pyknosis), loss of hepatocellular borders, and areas of necrosis. All paraffin sections of liver biopsy were examined for apoptosis using the transferase-mediated deoxyuridine triphosphate nick-end labeling method (TUNEL, In Situ Cell Detection Kit, Roche Biochemicals, Mannheim, Germany). The number of TUNEL-positive cells was calculated in 6 random high-powered (magnification: 200x) microscopic fields. The apoptosis index was defined as the number of apoptotic cells in every hundred and counted cells using Image-Pro-Plus® Software.

### 2.6. Mesurement of TNF-*α* and IL-6 by Quantitative Real-Time PCR

RNA was extracted by TRIzol reagent (Invitrogen, Carlsbad, CA). The total RNA was isolated to synthesize first-strand cDNA by using an RT-PCR kit (Invitrogen, Carlsbad, CA, USA). The primers of TNF-*α* and IL-6 in this study were as follows: TNF-*α* forward 5-CAT GAT CCG AGA TGT GGA ACT GGC-3, reverse 5-CTGGCTCAGCCACTCCAG C-3.IL-6, forward 5^'^–TCCTACCCCAACTTCCAATGCTC–3^'^, and reverse 5^'^–TTGGATGGTCTTGGTCCTTAGCC–3^'^. GAPDH was used as a control in a similar way by using the following primers: forward5^'^–CCACAGTCCATGCCATCAC–3^'^, reverse 5^'^–TCCACCACCCTGTTGCTGTA–3^'^.

### 2.7. c-Fos Immunofluorescence

The rats were perfused with phosphate buffered saline (PBS) and paraformaldehyde after ischemia-reperfusion, followed by the collection and postfixation of the brains. Subsequently, we transferred the specimens to 0.1 M phosphate buffer containing 30% sucrose and kept them at 4°C for 72 hours. Twenty-five-micrometer sections through the DMV were cut in a frozen section machine. The sections were washed twice with PBS, then blocked with 3% BSA for 2 hours, and incubated with anti-c-Fos antibody (dilution 1 : 100, Santa Cruz, USA) overnight at 4°C. Repeat the washing with PBS for three times. After that, incubate sections with a second antibody at room temperature for 2 hours. The samples were then observed under an immunofluorescent microscope.

### 2.8. Microinjection into DVC

Animals were anesthetized by intraperitoneal injection of pentobarbitone (60 mg·kg^−1^, Dainabot, Osaka, Japan) and fixed in a stereotactic instrument with the head put between the lambda and bregma. Microsyringes were customized with a 0.3-millimeter external diameter. Each rat was microinjected slowly with either 1 *μ*L of NS or 1 *μ*g remifentanil diluted into 1 *μ*L of normal saline into DVC (0.4 millimeter lateral to the midline, 7.9 millimeter below the cranial surface and at occipital crest). Mechanical ventilation was prepared for potential respiratory depression. The dosage of remifentanil was set at 1 *μ*g because our preliminary experiment indicated that it will not induce respiratory depression during a very short time. 5 minutes after injection, the needles were withdrawn slowly.

### 2.9. Statistical Analysis

Data were analyzed with a personal computer statistical software package (Prism version 5.0; GraphPad Software, San Diego, CA). Results are expressed as the mean ± SEM. Student's *t*-test and chi-square test were applied to compare values between groups. Statistical differences were considered significant if *P* value was less than 0.05. All *P* values are the results of two-sided tests.

## 3. Results

A total of 104 rats were used in this study, and 2 rats were excluded because of breath suppression and 1 rat for unexplained bleeding during the surgical procedure.

### 3.1. Effect of Blocking Peripheral *μ*-Opioid Receptor on RPC following HIR

**Figure 2:**
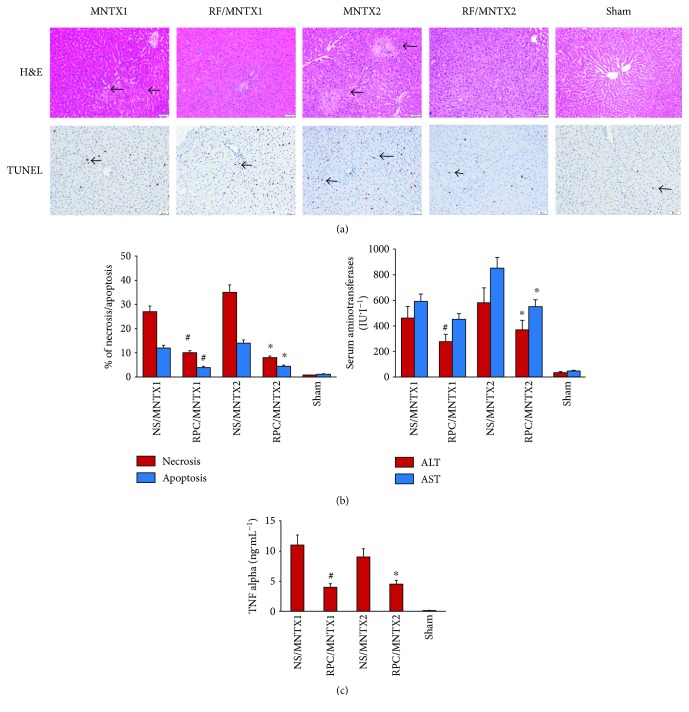
Effect of opiate agonist on hepatic ischemia reperfusion when peripheral opioid receptors were antagonized. Rats were divided into 5 groups, and animals were performed with laparotomy and dissection of the portal vein but not clamping in the sham group, or treated with MNTX1 (0.5 mg·kg^−1^) or MNTX2 (5 mg·kg^−1^) into the peritoneal cavity, followed by an intravenous injection of 2 *μ*g·kg^−1^·min^−1^ of remifentanil or normal saline within 15 minutes. Hepatic ischemia reperfusion (IR) was induced 10 minutes after the treatment above with ischemia for 45 minutes and 120-minute reperfusion. *n* = 8 in each treatment group and *n* = 5 in the sham group. (a) Hepatic tissue histologic changes were processed with hematoxylin and eosin (H&E) staining for light microscopy examination. Photograph depicts a typical pattern of focal necrosis (black arrows) after ischemic degeneration of hepatocytes around the central venous area. Hepatocyte apoptosis was determined by TUNEL staining. Photograph depicts a typical pattern of apoptotic cells (black arrows). Areas of necrosis and percentage of apoptotic cells were significantly decreased in the remifentanil preconditioning (RPC) groups than in the normal saline (NS) groups (magnification: 200x; ^#^*P* < 0.05 versus NS/MNTX1; ^∗^*P* < 0.05 versus NS/MNTX2). (b) Serum alanine aminotransferase (ALT) was significantly lower in the RPC/MNTX1 groups than in the NS/MNTX1 groups, while serum alanine aminotransferase (ALT) and aspartate aminotransferase (AST) concentrations were significantly lower in the RPC/MNTX2 groups than in the NS/MNTX2 groups (^#^*P* < 0.05 versus NS/MNTX1; ^∗^*P* < 0.05 versus NS/MNTX2). (c) The serum level of TNF-*α* significantly decreased in the remifentanil preconditioning (RPC) groups than in the NS groups (^#^*P* < 0.05 versus NS/MNTX1; ^∗^*P* < 0.05 versus NS/MNTX2).

Before remifentanil preconditioning and the HIR model being established, animals were intraperitoneally pretreated with MNTX, a peripheral antagonist. When preinjected with either low (0.5 mg·kg^−1^) or higher (5 mg·kg^−1^) dose of MNTX, the hepatic injury was significantly lower in both RPC groups compared in the NS group, which was highlighted by H&E staining as well as apoptotic cell calculation on TUNEL staining (Figure 2(a), ^#^*P* < 0.05 versus NS/MNTX1; ^∗^*P* < 0.05 versus NS/MNTX2). Meanwhile, the serum concentration of both aspartate transaminase (AST) and alanine transaminase (ALT) was statistically lower in the RPC/MNTX2 group, and ALT was decreased in the RPC/MNTX1 group compared with the NS/MNTX1 group (Figure 2(b), ^#^*P* < 0.05 versus NS/MNTX1; ^∗^*P* < 0.05 versus NS/MNTX2). Besides, serum levels of TNF-*α* was significantly decreased in both RPC groups (Figure 2(c), ^#^*P* < 0.05 versus NS, NS/Va, and RPC/Va groups). The sham group showed no liver injury since liver aminotransferases (ALT and AST), TNF-*α*, and cellular apoptosis were all kept in a very limited level.

### 3.2. Intravenous Remifentanil Activates Dorsal Motor Nucleus of the Vagus (DMV)

**Figure 3:**
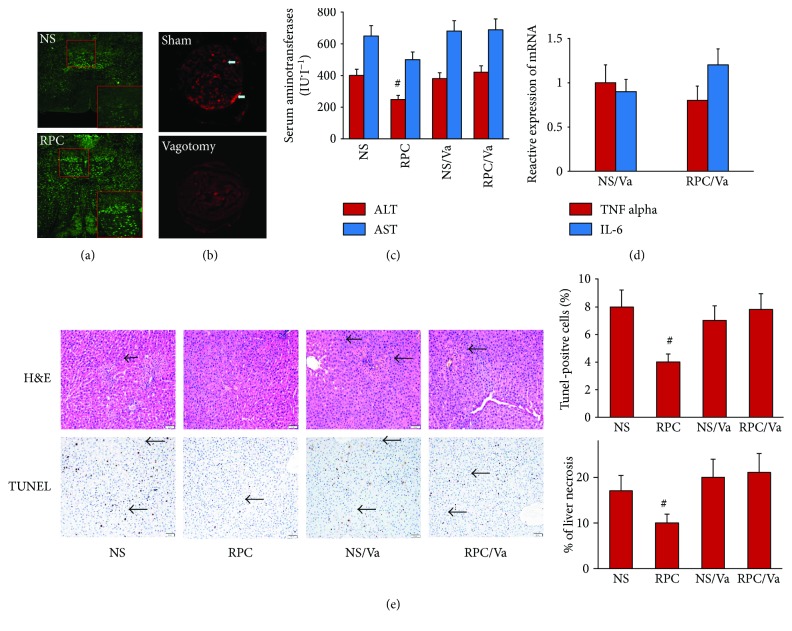
Hepatic protective effect of opiate agonist disappeared after vagotomy. Rats were divided into 4 groups with or without vagotomy and then treated with an intravenous injection of remifentanil (2 *μ*g·kg^−1^·min^−1^) or normal saline (NS) within 15 minutes. Hepatic IR was induced 10 minutes after the treatment above with ischemia for 45 minutes and 120-minute reperfusion. *n* = 8 in each group. (a) Activation of neuron was manifested by c-Fos immunofluorescence. Red frame regions revealed that remifentanil preconditioning (RPC) activated DMV compared with the NS group. (b) Fluorescent Dil tracer indicated that the communication of the vagus nerve between the liver and the inferior ganglion of the vagus nerve in the vagotomized group was almost interdicted, so that the operation of vatogomy was reliable. (c) Serum alanine aminotransferase (ALT) was significantly lower in the RPC group than in the NS group and vagotomy groups. (^#^*P* < 0.05 versus NS, NS/Va, and RPC/Va groups). (d) There was no statistical significance in relative mRNA expression of TNF-*α* and IL-6 from quantitative real-time PCR between the NS/Va and RPC/Va groups. (e) Photograph depicts a typical pattern of focal necrosis after ischemic insult. Black arrows indicate ischemic changes that were seen less seriously in the remifentanil preconditioning (RPC) group compared with the other groups. The degree of apoptosis and the number of terminal deoxynucleotide transferase-mediated deoxyuridine triphosphate nick-end labeling- (TUNEL-) positive cells (black arrows) were also decreased in the remifentanil preconditioning (RPC) group (magnification: 200x; ^#^*P* < 0.05 versus NS, NS/Va, and RPC/Va groups).

10 minutes after caudal venous injection of 2 *μ*g·kg^−1^·min^−1^ of remifentanil, the brain stem was prepared to perform c-Fos staining, which presented a highlight green fluorescence area in the brain stem (Figure 3(a)), and it was the dorsal motor nucleus of the vagus (DMV) and confirmed that central vagus nuclei was activated by intravenous injection of remifentanil.

### 3.3. Selective Hepatic Vagotomy Abolishes the Protection of Intravenous RPC on Liver IRI

The retrograde tracing of the vagus nerve was labelled by fluorescence, and the analysis of the dissected inferior ganglion of the vagus nerve showed that the selective hepatic vagotomy was successful since the fluorescent tracer was nearly absent in the inferior ganglion of the vagus nerve of vagotomized animals when compared with nonvagotomized animals (Figure 3(b)). However, there was no significant difference between the RPC/Va and NS/Va groups of the serum concentration of AST and ALT and inflammatory cytokines (TNF-*α* and IL-6) as well as the hepatic histologic damage and apoptosis (*P* > 0.05, Figures 3(c)–3(e)).

### 3.4. Remifentanil Microinjection into DVC Attenuates Liver IRI, Which Was Reversed by Selective Hepatic Vagotomy

**Figure 4:**
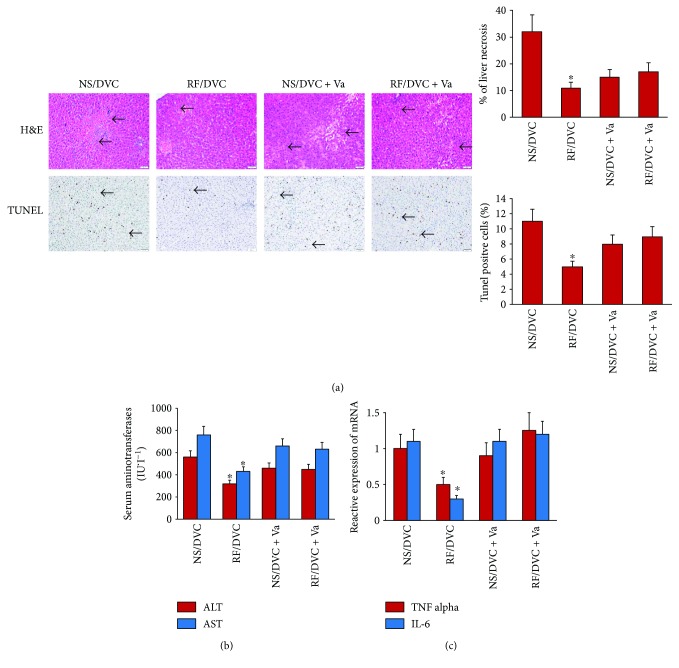
Microinjection of remifentanil in DVC alleviated hepatic ischemia reperfusion injury but abolished by vagotomy. Rats were divided into 4 groups with or without vagotomy and then injected with 1 *μ*g of remifentanil or normal saline into DVC. Hepatic IR was induced 10 minutes after the treatment above with ischemia for 45 minutes and 120-minute reperfusion. *n* = 8 in each group. (a) Photograph depicting a typical pattern of focal necrosis (black arrows) after ischemic degeneration of hepatocytes around the central venous area showed that histologic damage was significantly decreased when injected with remifentanil into DVC (RF/DVC) but not in the NS/DVC and RF/DVC + Va groups. Hepatocyte apoptosis determined by TUNEL staining indicated that hepatocyte apoptosis was significantly decreased in the group with DVC injection of remifentanil (RF/DVC) without vagotomy (magnification: 200x; ^∗^*P* < 0.05 versus NS/DVC, NS/DVC + Va, and RF/DVC + Va groups). (b) Serum alanine aminotransferase (ALT) and aspartate aminotransferase (AST) were significantly decreased in the group with DVC injection of remifentanil (RF/DVC) than in the NS/DVC and RF/DVC + Va groups (^∗^*P* < 0.05 versus NS/DVC, NS/DVC + Va, and RF/DVC + Va groups). (c) TNF-*α* and IL-6 mRNA expression measured by quantitative real-time PCR were lower in the group injected with remifentanil into DVC (RF/DVC) compared with the NS/DVC and RF/DVC + Va groups (^∗^*P* < 0.05 versus NS/DVC, NS/DVC + Va, and RF/DVC + Va groups).

Hepatic histologic injury, apoptotic liver cells, ALT, and AST, as well as the inflammatory cytokines (TNF-*α* and IL-6), were all decreased following microinjection of remifentanil into the DVC in the brain stem (Figure 4, ^∗^*P* < 0.05 versus NS/DVC, NS/DVC + Va, and RF/DVC + Va groups). Nevertheless, when animals were selectively vagotomized, microinjection of remifentanil into DVC did not change the serum level of aminotransferases, apoptotic liver cell number, tissue injury, or inflammatory cytokine levels.

## 4. Discussion

Many studies showed that remifentanil protects the liver from IRI in animal models, which is in accordance with our published study [11]. However, the underlying mechanism has not been elucidated yet. In this way, our study showed that when pretreating rats with high dose or low dose of peripheral *μ*-opioid antagonist, RPC can still attenuate liver IRI, implying that peripheral *μ*-opioid receptor is not the only mediator of the RPC protective pathway against hepatic IRI. The RPC protection was even abolished once the hepatic vagus nerve was selectively cut off, which was showed with retrograde fluorescence tracer technique by Dil. Therefore, the vagus nerve should be a crucial regulating pathway by which RPC exerts a hepatic protective role against IRI.

Intravenous injection of remifentanil activated DMV in the brain stem, indicating that remifentanil interacts with the central parasympathetic system and DMV is the primary reactor. Subsequently, when remifentanil was preconditioned by a direct microinjection into DVC, the protection against liver IRI reappeared; however, it was abolished by a selective liver vagotomy. Hence, our study concluded that a central vagal pathway, instead of peripheral *μ*-opioid receptor (MORs), is primarily participated in the protective role of RPC against liver IRI.

Remifentanil, a highly selective *μ*-opioid receptor agonist, shows protective effects against ischemic injury in multiple organs, such as the liver [26, 27], heart [28, 29], and small intestine [30, 31] in animals. Our previous study showed that remifentanil preconditioning attenuates hepatic IRI by increasing the expression of iNOS [11]. However, the precise mechanism underlying the production of iNOS has not been elucidated yet. Remifentanil penetrates the liposoluble BBB once systemically administered, due to its special pharmacokinetic profiles; therefore, there might be stimulated and elicit various signal pathways once it was systemically administrated [32]. Another study showed that preconditioning by intracerebral ventricular injection with morphine endows protection against myocardial IRI via increasing the expression of calmodulin (CaM) in the hippocampus [33]. Therefore, it is plausible that the central neuronal pathways participate in the protective function of opioid preconditioning against hepatic IRI. Our study confirmed the hypothesis, revealing that neurons in DMV were activated following peripheral remifentanil pretreatment. Moreover, microinjection with remifentanil into DVC still conferred hepatic protection during IRI, which can be reversed by selective hepatic vagotomy, implying that the hepatic vagus nerve was the crucial pathway in the RPC-associated liver protection against IRI.

DVC, a collection of three adjacent nuclei in the caudal, dorsomedial medulla oblongata of the brain, is abundant of MORs. The activation of these MORs at the level of DMV motor neurons has been shown to affect gastric functions [34]. The DVC can not only integrate the information from the peripheral organs and innervate viscera via the vagus nerve, but also plays a crucial role in energy balance and immune regulation. Evidence showed that DVC modulates liver function directly, including glucose production [35] and secretion of triglyceride-rich lipoproteins [36]. An activation of neurons in DVC has also been showed to be effectively protecting the intestine from IRI [37], so it may also regulate IRI in the liver. Our study confirmed this hypothesis, showing that microinjection of remifentanil into DVC protects animals from hepatic IRI. We have used 1 *μ*g remifentanil since our pilot study, and a dosage of 1 *μ*g was the most effective dosage without significant undesirable effects, such as respiratory depression. However, mechanical ventilation was also prepared to avoid such secondary effects.

Most publications studying opioid receptors in hepatic IRI are mainly focused on the liver itself [38]. Nevertheless, our study suggested an involvement of central parasympathetic pathway implicated in hepatic IRI for the first time, which is in accordance with our preliminary observation that naloxone, the *μ*-opioid receptor antagonist, cannot abolish the induced protection by remifentanil [15]. The activated vagus neurons are identified to confer cardioprotection in a remote precondition model [39]. Thus, opioid preconditioning and remote preconditioning may share the same pathway, in which the vagus nerve plays an essential role. The vagus nerve innervates most vital organs such as the heart, liver, and intestine and elicits the anti-inflammation pathways [40], which is one of the pathological components underlying ischemia-reperfusion injury and could be modulated or suppressed by the vagus nerve activation. Studies proved that vagal activation protects the stomach from IRI [41], as well as decreases Fas-induced hepatocyte apoptosis [22]. Vagal activation which was initiated during ischemia process, rather than reperfusion process, exerts cardioprotection in ischemia-reperfusion [42]. Our research highlighted that remifentanil alone can also activate DMV, so it probably activates the central vagus nerve and modulates the inflammatory reaction at a relatively low level, which inhibits the succedent inflammatory cascade induced by IRI.

Our study investigated a central neuronal pathway in remifentanil preconditioning against hepatic IRI; however, other mechanisms such as peripheral pathways may also be implicated. We could not exclude the peripheral pathway contributing to remifentanil precondition against hepatic ischemia-reperfusion; therefore, further studies are warranted to illustrate the precise mechanism underlying protective effects of remifentanil preconditioning against hepatic IRI.

In conclusion, our study demonstrated that remifentanil preconditioning provides protection against hepatic IRI by activating the central vagus nerve instead of the peripheral *μ*-opioid receptors.

## 5. Conclusions

Remifentanil preconditioning attenuates hepatic ischemia-reperfusion injury in rats via neuronal activation in the DVC.
